# *Entamoeba histolytica* Cyclooxygenase-Like Protein Regulates Cysteine Protease Expression and Virulence

**DOI:** 10.3389/fcimb.2018.00447

**Published:** 2019-01-09

**Authors:** Preeti Shahi, France Moreau, Kris Chadee

**Affiliations:** Department of Microbiology, Immunology and Infectious Diseases, Snyder Institute for Chronic Diseases, University of Calgary, Calgary, AB, Canada

**Keywords:** *Entamoeba histolytica*, parasites, cox like protein, cysteine protease, actinin like protein, virulence, pro-inflammatory cytokines

## Abstract

The intestinal protozoan parasite *Entamoeba histolytica* (*Eh*) causes amebiasis associated with severe diarrhea and/or liver abscess. *Eh* pathogenesis is multifactorial requiring both parasite virulent molecules and host-induced innate immune responses. *Eh*-induced host pro-inflammatory responses plays a critical role in disease pathogenesis by causing damage to tissues allowing parasites access to systemic sites. *Eh* cyclooxygenase (*Eh*Cox) derived prostaglandin E_2_ stimulates the chemokine IL-8 from mucosal epithelial cells that recruits neutrophils to the site of infection to exacerbate disease. At present, it is not known how *Eh*Cox is regulated or whether it affects the expression of other proteins in *Eh*. In this study, we found that gene silencing of *Eh*Cox (*EhCoxgs*) markedly increased endogenous cysteine protease (CP) protein expression and virulence without altering CP gene transcripts. Live virulent *Eh* pretreated with arachidonic acid substrate to enhance PGE_2_ production or aspirin to inhibit *Eh*Cox enzyme activity or addition of exogenous PGE_2_ to *Eh* had no effect on *Eh*CP activity. Increased CP enzyme activity in *EhCoxgs* was stable and significantly enhanced erythrophagocytosis, cytopathic effects on colonic epithelial cells and elicited pro-inflammatory cytokines in mice colonic loops. Acute infection with *EhCoxgs* in colonic loops increased inflammation associated with high levels of myeloperoxidase activity. This study has identified *Eh*Cox protein as one of the important endogenous regulators of cysteine protease activity. Alterations of CP activity in response to Cox gene silencing may be a negative feedback mechanism in *Eh* to limit proteolytic activity during colonization that can inadvertently trigger inflammation in the gut.

## Introduction

*Entamoeba histolytica* (*Eh*) is an invasive extracellular protozoan parasite responsible for amebic colitis and liver abscess (World Health Organization, 1998). It is one of the major cause of severe diarrhea in areas of poor sanitation and nutrition particularly in tropical and developing countries. While the majority of *Eh* infection remains asymptomatic, about 10% of infection converts to an invasive phenotype where *Eh* invades the mucosal epithelium resulting in 100,000 death/year (World Health Organization, 1997; Stanley, 2003).

The host innate immune status and parasite virulence factors play major roles in disease pathogenesis (Faust and Guillen, 2012; Verkerke et al., 2012; Marie and Petri, 2014; Nozaki and Bhattacharya, 2015). The host immune response during *Eh* invasion of the colonic mucosa is characterized by increased levels of pro-inflammatory cytokines that recruits inflammatory cells including macrophages and neutrophils (Seydel et al., 1997; Mortimer and Chadee, 2010; Nakada-Tsukui and Nozaki, 2016) to the site of infection. The major *Eh* virulent factors identified to date are the galactose/N-acetyl-D-galactosamine (Gal/GalNAc) lectin (Gal-lectin), cysteine proteinases, amoebapore, and prostaglandin E_2_ (Moonah et al., 2013; Marie and Petri, 2014).

Prostaglandins are lipid mediators synthesize from arachidonic acid through cyclooxygenase and are associated with various diarrheal disease including bacterial and inflammatory bowel diseases (Ahrenstedt et al., 1994; Alcantara et al., 2001; Resta-Lenert and Barrett, 2002). We have shown that *Eh* synthesizes PGE_2_ through a cyclooxygenase like enzyme as confirmed by gas chromatography/mass spectrometry analysis (Belley and Chadee, 2000). Surprisingly, *Eh*PGE_2_ was not immunosuppressive to reduce host defenses but rather, PGE_2_ bound EP4 receptors on colonic epithelial cells and stimulated the potent neutrophil chemoattractant, IL-8 mRNA expression and protein production. In addition, *Eh*PGE_2_ also altered tight junction proteins and increased ion permeability that led to diarrhea in intestinal amebiasis (Lejeune et al., 2011). Even though *Eh* produces high levels of PGE_2_ in the presence of arachidonic acid the parasite can also stimulate host cells such as macrophages and colonic epithelial cell to produce PGE_2_ as part of the pro-inflammatory response elicited by the parasite (Stenson et al., 2001; Sanchez-Ramirez et al., 2004). *Eh*Cox encodes a functional cyclooxygenase enzyme as evidenced by Cox enzymatic assays. An unusual aspect is that *Eh*Cox is primitive and has little homology to other Cox proteins from mammalian cells and some eukaryotes. At present, the biological function of *Eh*Cox other than being an enzyme that catalyzes the production of PGE_2_ in *Eh* is not known.

In this study, we made the seminal observation that silencing *Eh*Cox enhanced cysteine protease protein expression and enzyme activity independent of *Eh*Cox-induced PGE_2_ production. These results suggest that increased cysteine protease activity in *EhCoxgs* is linked to increase parasite-induced inflammation and pathogenicity. These findings increase our understanding on the molecular basis of pathogenicity in *Eh* and how dissimilar enzymes can regulate their activity in the parasite.

## Materials and Methods

### Reagent

E64, leupeptin, aprotinin, and Nonidet P-40 detergent were obtained from Sigma-Aldrich. Z-VVR-AMC substrate was purchased from Enzo Life Sciences. The Z-Arg-Arg-pNA.2 HCl substrate was purchased from Bachem. Mouse monoclonal anti-actin clone C4 antibody was purchased from MP Biomedical, LLC. Antibodies to *Eh*CP4 and the CP inhibitors WRR483 and WRR605 were a kind gift from Dr. Sharon Reed, University of California, San Diego. *Eh*CP5 and *Eh*Cox1 like antibodies were generated in rabbits using recombinant proteins expressed in *E. coli* (Belley and Chadee, 2000). Ubiquitin antibody (P4D1) was from Cell Signaling Technology and cycloheximide from Sigma-Aldrich.

### Cultivation and Harvesting of *E. histolytica*

G3 *Eh* were grown axenically in TYI-S-33 medium at 37°C. After 72 h, logarithmic-growth-phase *Eh* cultures were harvested by chilling on ice for 9 min, pelleted at 200 g, and washed two times with PBS. For the detection of proteins and enzymatic activity, *Eh* lysate was prepared by using three cycles of freeze-thawlysis and proteins quantified by the bicinchoninic acid protein assay, using bovine serum albumin as protein standard (#23225, Thermo Scientific). *Eh* secretory protein (SP) were prepared as described previously (Lidell et al., 2006). Briefly, secreted components were collected from mid-log phase *Eh* incubated in Hanks' balanced salt solution (Invitrogen) for 2 h at 37°C at a final concentration of 2 × 10^7^ *Eh* per ml. Following incubation, *Eh* was removed by centrifugation at 10,000 × g for 10 min. Secretory proteins were quantified by the bicinchoninic acid protein assay. To quantify the growth of control and *EhCoxgs*, 2.5 × 10^5^ log phase *Eh* were inoculated in 14 ml TYI-S-33 medium and the number of parasites counted every 24 h using a hemocytometer.

### Cloning of the *Eh*Cox-Like Gene

*Eh*Cox-like gene (Acc No. AF208390) 500 bp long 5′ end of protein-coding region was amplified by PCR from cDNA using specific primer containing stu1 and sac1 restriction sites (Table 1). The PCR product was sub cloned using the pGEM-T Easy vector system (Promega) and then digested with the restriction enzymes, stu1 and sac1. The digested DNA insert was cloned into StuI- and SacI-digested pSAP2-gunma (kind gift from Dr. Tomoyoshi Nozaki, Tokyo, Japan). This construct was verified by sequencing. G3 *Eh* were harvested during mid-log growth and transfected with a silencing plasmid (pSAP2-gunma-cox) using the Lipofectamine (Life technologies) and OPTIMEMI medium (Life Technologies) supplemented with 5 mg/ml L-cysteine and 1 mg/ml ascorbic acid (transfection medium) and pH 6.8 as previously described (Fisher et al., 2006) Transfected *Eh* were selected with G418 (Sigma) over a 3-week period, starting with 6 μg/ml and ending with 24 μg/ml. Silencing was assessed in these strains using quantitative reverse transcription-PCR (qRT-PCR) and western blot analysis. Once gene silencing was confirmed, the G418 selection was removed, and then silencing was again confirmed and quantified.

**Table 1:** Primers used in this study.

	**Name**	**Sequence 5′-3′**	**Annealing temp (°C)**
*E. histolytica*	*Eh*Cox	Fwd:5′ stu1: AGGCCTATGACTGGAAATAAAGAAT	55
		Fwd: 3′ sac1: GAGCTCGAAACTGCTTCTGTTA	
	*Eh*Cox RT	Fwd: TGACTGGAAATAAAGAATGGGA	59
		Rev: CCATAAGACTAATCAAAATATCTGACT	
	CP5 RT	Fwd: AATTCATGGGGAACTATTTGG	59
		Rev: CATCAGCAACCCCAACTGG	
	CP4 RT	Fwd: GTTAACCATGGTGTTGCCGCTGTA	59
		Rev: GCATCATGAGCACCAGTTGGGAAA	
	CP1 RT	Fwd: ATAAACACTTCACAGCAGTTGA	59
		Rev: TTCTTCATTTGTCATAGCAGC	
	CP2 RT	Fwd: ATCCAAGCACCAGAATCAGT	59
		Rev: TTCCTTCAAGAGCTGCAAGT	
	rDNA RT	Fwd: TCAAAAAGCAACGTCGCTA	59
		Rev: AGCCCGTAAGGTGATTTCT	
Murine	IL-1β	Fwd: GCCTCGTGCTGTCGGACCCA	58
		Rev: CTGCAGGGTGGGTGTGCCGT	
	TNF-α	Fwd: ATGAGCACAGAAAGCATGATC	60
		Rev: TACAGGCTTGTCACTCGAATT	
	IFN-γ	Fwd: TCAAGTGGCATAGATGTGGAAGAA	58
		Rev: TGGCTCTGCAGGATTTTCATG	
	Actin	Fwd: CTACAATGAGCTGCGTGTG	58
		Rev: TGGGGTGTTGAAGGTCTC	
	KC	Fwd: ACCCAAACCGAAGTCATAGC	60
		Rev: TCTCCGTTACTTGGGGACAC	

### Preparation of *E. histolytica* Nuclear Proteins (*Eh*NP)

*Eh* were washed twice in PBS and suspended in lysis buffer [100 mM Tris (pH 7.4), 1 μg/ml E-64 (Sigma), 2 μg/ml leupeptin, 7.4 μg/ml aprotinin and 0.5% Nonidet P-40 detergent] on ice for 15 min. Nuclei were pelleted by centrifugation at 2,000 × g for 15 min, washed with lysis buffer, and suspended in 0.1 M sodium phosphate buffer (pH 7.0). The protein concentration was determined by the bicinchoninic acid method using bovine serum albumin as protein standard (Thermo Scientific).

### Quantitative Real-Time (qRT) PCR-Based Analysis of Gene Expression

Total RNA was extracted from logarithmic-growth-phase *Eh* using a Trizol reagent method (Invitrogen; Life Technologies, Burlington, ON) and the yield and purity determined by the ratio of absorbance at 260/280 nm (NanoDrop, Thermo Scientific). DNase I-treated total RNA was used in the RT reaction using qScript cDNA Synthesis kit and PerfeCTa SYBR Green Supermix (Quantabio). Real-time qPCR was performed using a Rotor Gene 3000 real-time PCR system (Corbett Research). The PCR reaction mix (20 μl) comprised 1x SYBR Green, 25 ng cDNA and 1 μM of primers. A complete list of the primer sequences and conditions used are listed in Table 1. Results were analyzed using the 2-ΔΔCT methods and expressed as fold changes.

### Sample Preparation for Proteomics

*Eh* lysates were prepared as described before. After proteins precipitation with TCA, the supernatant was discarded and proteins were air dried and suspended in 200 μL of urea/HCl. The sample was then treated with 10 mM of dithiothreitol and incubated at 37°C for 30 min and transferred into Microcon YM-30 (Millipore) and centrifuge at 14,000 × g at 20°C for 15 min. The eluates were discarded, 200 μL of UA was pipetted into the filtration unit, and the units were centrifuged again. Then 100 μL of 0.05 M iodoacetamide in Urea/Tris was added to the filters, and samples were incubated in darkness for 20 min. Filters were washed twice with 100 μL of Urea/Tris and, with 100 μL of 0.05 M ammonium bicarbonate at 14,000 × g for 15 min. Proteins were digested in 40 μL of trypsin in 0.05 M ammonium bicarbonate at 37°C overnight. The released peptides were collected by centrifugation at 14,000 × g for 10 min followed by two washes with 0.05 M, 40 μL ammonium bicarbonate and with 40 μL of 0.05 M NaCl. After isolation of the peptides, samples were transferred into new Eppendorf tube.

### LC-MS/MS Analysis

Total protein and peptides content were analyzed on an Orbitrap Fusion Lumos Tribrid mass spectrometer (Thermo Scientific) operated with Xcalibur (version 4.0.21.10) and coupled to a Thermo Scientific Easy-nLC (nanoflow Liquid Chromatography) 1,200 system. Isolated trypsin treated peptides (2 μL) were loaded onto an Easy Spray Column (ES803) at a maximum of 700 bars (2 μm particle column). Further, peptides were eluted using a 120 min gradient from 5 to 40% (5 to 28% in 105 min followed by an increase to 40% B in 15 min) of 0.1% formic acid in 80% LC-MS grade acetonitrile at a flow rate of 0.3 μL/min and separated on a C18 analytical column (ES803). Then, peptides were electrosprayed using 2.0 kV voltages into the ion transfer tube (300°C) of the Orbitrap Lumos operating in positive mode. A full MS scan was performed by Orbitrap at a resolution of 12,0000 FWHM to detect the precursor ion having a *m*/*z* between 375 and 1575 and a +2 to +7 charge. The Orbitrap AGC (Auto Gain control) and the maximum injection time were set at 4e5 and 50 ms, respectively. The Orbitrap was working with a 3 s cycle time for precursor selection and most intense precursor ions presenting a peptidic isotopic profile, having an intensity threshold of at least 5,000 were isolated using the quadrupole and fragmented with HCD (30% collision energy) in the ion routing multipole. The fragment ions (MS^2^) were analyzed in the ion trap at a rapid scan rate. The AGC and the maximum injection time were set at 1e4 and 35 ms, respectively, for the ion trap. Dynamic exclusion was enabled for 45 s to avoid of the acquisition of same precursor ion having a similar m/z (plus or minus 10 ppm).

### Database Search

With the help Raw Converter (v1.1.0.18; The Scripps Research Institute), raw data files (^*^.raw) were converted into Mascot Generic Format (MGF). Monoisotopic precursors having a charge state of +2 to +7 were selected for conversion. The mgf file was used to search a database by using Mascot algorithm (Matrix Sciences; version 2.4). Search parameters for MS data included trypsin as enzyme, a maximum number of missed cleavage of 1, a peptide charge equal to 2 or higher, cysteine carbamidomethylation as fixed modification, methionine oxidation as variable modification and a mass error tolerance of 10 ppm. A mass error tolerance of 0.6 Da was selected for the fragment ions. Only peptides identified with a score having a confidence higher than 95% were kept for further analysis. The Mascot dat files were imported into Scaffold (v4.3.4, Proteome Software Inc.) for comparison of different samples based on their mass spectral counting.

### Western Blots

Proteins (30 μg) were loaded on 12% SDS-PAGE gel followed by transfer onto polyvinylidene fluoride (PVDF) membrane and blocking in 5% skim milk. Blots were then probed with 1:5,000 *Eh*Cox antibody or 1:1,000 *Eh*CP5 antibody/1:500 *Eh*CP4 antibody/1:1,000 Actin antibody for 16 h at 4°C. After incubation with one of the previously described primary antibodies, the blots were incubated with appropriate HRP-conjugated secondary antibody for 1 h at RT, and then developed using ChemiLucent ECL detection (EMD Millipore).

### *Eh*Cox Activity Assay

*Eh*COX activity assay was performed on nuclear fractions of *Eh* (*Eh*NP). *Eh*NP (100 μg) was incubated for 30 min with 1 mM aspirin (ASA) followed by 100 μM arachidonic acid (AA) or vehicle for 1 h at 37°C in sodium phosphate buffer containing 200 μM tryptophan and 2 μM hematin (Sigma) in 500 μl volume. After centrifugation, PGE_2_ in the supernatant was extracted with Amprep C2 ethyl columns (Amersham Biosciences) following manufacturer's protocol. PGE_2_ was quantified by using an enzyme immunoassay kit (Cayman Chemical, Ann Arbor, MI).

### *Eh* Cysteine Proteinase Activity

*Eh*CP5 and *Eh*CP4 activity in lysate and secretory protein was determined using known chromophoric substrate benzyloxycarbonyl-L-arginyl-L-arginine p-nitroanilide (Z-Arg-Arg-pNA) and fluorogenic substrate benzyloxycarbonyl-L-val-L-val- 7-amino-4-methylcoumarin (Z-VVR-AMC), respectively, as previously described (Leippe et al., 1995; He et al., 2010). Briefly, substrate was incubated for 0–20 min at 37°C with either *Eh* lysate/secretory proteins (50 μg) alone or pretreated with CP inhibitor-E64 [L-trans-epoxysuccinyl-leucyl-amido-(4-guanidino) butane]/CP-1(WRR483) and CP-4 (WRR605) inhibitors. Cleavage of the chromophoric (Z-Arg-Arg-pNA) and fluorogenic (Z-VVR-AMC) substrate were detected at the 405 and 460 nm wavelength, respectively.

### Gelatinase Gel Substrate Gel Electrophoresis

For analysis of protease activity by gelatinase substrate gel electrophoresis, 12% SDS polyacrylamide was copolymerized with 0.1% gelatin as described previously (Hellberg et al., 2000). Briefly, *Eh* lysate were prepared by three freeze-thaw cycles in HBSS and centrifuged for 10 min at 10,000 × g. Supernatant was separated on 0.1% gelatin copolymerized 12% SDS PAGE. After separation of proteins, SDS was removed by two washings in 2.5% Triton X-100 for 1 h at room temperature. Gel was then incubated in developing buffer (20 mM DTT, 100 mM sodium acetate, pH 4.2, and 1% Triton X-100) at 37°C for 3 h. The gel was stained with Coomassie blue. Clear band represent cysteine protease activity.

### *Eh* Erythrophagocytosis Assay

Fresh human erythrocytes were obtained in DPBS and stained with Phicoerythrin by using PKH26 Red Fluorescent cell linker kit (PKH26, Sigma-Aldrich). The erythrocytes were counted and used in 1:100 (*Eh:* erythrocyte) ratio. *Eh* and erythrocytes were incubated for 20 min at 37°C in DPBS. After interaction, cells were washed twice with DPBS and centrifuged at 3,000 × g for 5 min at 4°C. Lysis buffer (RBC lysis buffer, Sigma-Aldrich) was added for 1 min at RT followed by 0.5 ml addition of FBS and washed again with DPBS. The cells were fixed with 4% p-formaldehyde for 20 min at RT and washed with DPBS. To each sample a drop of fluoresave reagent was used for the slide preparation. The slides were observed under confocal microscope and fluorescent intensity was quantified by selecting region of interest (ROI) containing phagocytose erythrocyte and measuring the fluorescent intensity by using Image J software. The data was plotted as mean fluorescent intensity.

### *Eh* Cytopathic Assay

Caco-2 human colonic adenocarcinoma cells (ATCC, Manassas, VA) were grown to obtain confluent monolayers in DMEM medium (Invitrogen-Gibco) supplemented with 5% fetal bovine serum and 5 mg/ml penicillin-streptomycin under 5% CO_2_ at 37°C (Sigma-Aldrich). *Eh* disruption of a Caco-2 cell monolayer was determined using a previously described protocol (Belley et al., 1996). Briefly, *Eh* (10^5^/well) were incubated with Caco-2 cell monolayers in 24-wells tissue culture plates at 37°C. The incubation was stopped by placing the plates on ice and *Eh* were removed by washing with cold PBS. The Caco-2 cells that remained attached to the plates were stained with methylene blue (0.1% in 0.1 M borate buffer, pH 8.7). The dye was extracted from stained cells with 0.1 M HCl and color intensity of the extracted dye was measured spectrophotometrically at OD 660.

### Stability of *Eh*CP-A4 and *Eh*CP-A5 Protein

*Eh* and *EhCoxgs* were inoculated at 2.5 × 10^5^ and grown in TYI-S-33 media for 48 h before treatment with the protein synthesis inhibitor, cycloheximide at 100 μg/ml for 6, 12, and 24 h. After treatment *Eh* were harvested, wash in PBS and cell lysates were prepared in RIPA buffer 100 mM Tris-HCl, 150 mM NaCl, 2 mM EDTA, 0.1% sodium dexycholate pH 7.4 containing a protease inhibitor cocktail and 2 nM PMSF (Cruz-Vera et al., 2003). *Eh*CP protein remaining after treatment with cycloheximide was calculated as percentage protein remaining of levels at time zero (0 h). Protein half-life (t_1/2_) was calculated by linear regression analysis.

### Mice Colonic Loop Studies

Math1^GFP^ mice were purchased from Jackson laboratory and bred in-house. Colonic loops studies were done by inoculating live *Eh* suspended in 100 μL PBS (1 × 10^6^) into closed colonic loops as described previously (Belley and Chadee, 1999) This is a short-term infection model (3 h after infection). After 3 h, the colons were excised and tissue pro-inflammatory gene expression and myeloperoxidase activity was analyzed.

### Colonic Myeloperoxidase Activity Assay (MPO)

MPO activity was assayed in mouse colon samples as described previously (Kumar et al., 2017). Briefly, fresh frozen tissues were homogenized in 0.5% hexadecyltri-methylammonium bromide. Homogenized tissue was freeze-thawed three times, sonicated, and centrifuged at 10,000 g for 10 min at 4°C. Clear supernatant was collected and the reaction was initiated by addition of 1 mg/ml dianisidine dihydrochloride (Sigma, St. Louis, MO) and 1% H_2_O_2_, and change in optical density was measured at 450 nm.

### Math1 Expression via Non-invasive Whole-Body Imaging *ex vivo*

After 3 h of *Eh* infection, colons of Math1^GFP^ mice were surgically removed, imaged *ex vivo* using an *in vivo* Xtreme 4MP-imaging platform (Bruker, Billerica, MA, USA) to detect GFP expression. The imaging was performed in two steps. The first one is reflectance imaging (2 s exposure time) and the second one was with excitation at 470 nm and emission at 535 nm (5 s exposure time) i.e., fluorescent imaging. Images were acquired and analyzed from the *in vivo* Xtreme using Bruker molecular imaging software MI SE (version 7.1.3.20550). GFP expression in the colon was quantified by measuring the mean fluorescence (after background subtraction) in a constant ROI.

### Ethics Statements

All studies were carried out with the approval of the University of Calgary Animal Care Committee. Animal care committee have approved experimental procedure proposed and certifies that animal care was in accordance with recent policies by the Canadian council on Animal care.

### Statistical Analysis

Data was analyzed using Graphpad Prism 7 (Graph-Pad Software, San Diego, CA) for all statistical analysis. Student's *t*-test was used when two groups were compared. Statistical significance was assumed at *P* < 0.05.

## Results

### Silencing of the *Eh* Cyclooxygenase Like Gene Responsible for PGE_2_ Biosynthesis

**Figure 1:**
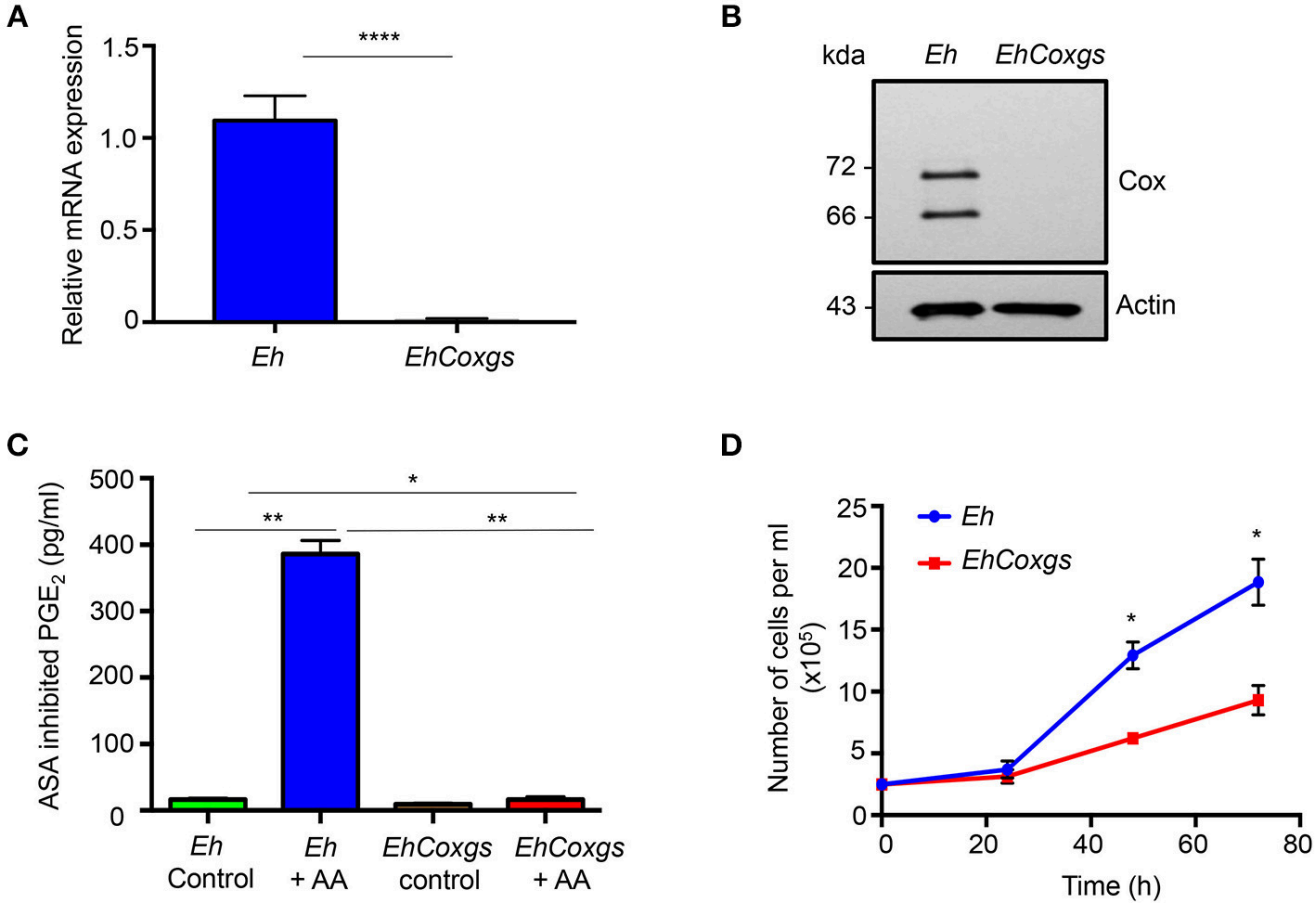
Silencing of the Cox-like protein in *E. histolytica*. **(A)** qPCR was used to monitor cox expression using cDNA from *E. histolytica*. The data indicate the changes in mRNA expression compared with controls. **(B)** Immunoblot blot analysis was performed on lysate prepared from control *Eh* and *EhCoxgs*. The proteins were separated on 8% SDS PAGE gels and analyzed with a polyclonal cox antibody or actin antibody. **(C)** PGE_2_ production was quantified using enzyme-linked immunosorbent assay kits following treatment of *Eh*NP with or without AA or aspirin (ASA) for 1 h at 37°C. The bars indicate the means and the error bars indicate the standard errors of the means for three different experiments. **(D)** Approximately 2.5 × 10^5^ control *Eh* and *EhCoxgs* in the logarithmic growth phase were inoculated into 14 mL fresh culture medium and *Eh* were counted every 24 h. Data shown are the means and the error bars indicate the standard errors of the means for three different experiments. The asterisks indicate the results of comparisons with the controls. ^*^P < 0.05, ^**^P < 0.01, ^****^P < 0.001. *Eh, E. histolytica*.

*Eh*-derived PGE_2_ not only induces pro-inflammatory IL-8 production but also disrupts colonic epithelial cell tight junction by coupling through EP4 receptors (Dey et al., 2003; Dey and Chadee, 2008; Lejeune et al., 2011). To analyze the biological functions of endogenous *Eh*Cox and *Eh*PGE_2_ mediated pro-inflammatory responses, we silenced the expression of the gene by small RNA-mediated transcriptional gene silencing in the G3 strain (Bracha et al., 2006). The specific gene repression was confirmed by reverse transcription PCR of corresponding cDNA and immunoblot analysis of proteins by using *Eh*Cox specific antibody. Complete silencing of *Eh*Cox was achieved in comparison to the *Eh* control (Figures 1A,B, Supplementary Figure 1). Immunoblot analysis detected the 72 and 66 Kda protein band in the lysate of control *Eh* as previously described (Dey et al., 2003). As predicted, *Eh*Cox enzymatic assay using nuclear fractions isolated from log-phase *Eh* showed almost no aspirin (ASA) inhibited PGE_2_ release (Dey et al., 2003) by *EhCoxgs* in comparison to control *Eh* incubated with arachidonic acid (AA) substrate (Figure 1C). We used ASA inhibited PGE_2_ release to accurately quantify PGE_2_ levels as *EhCoxgs* showed modest non-specific binding by enzyme immunoassay (EIA) that was not inhibited with ASA.

### Growth Kinetics of *EhCoxgs*

To determine whether *EhCoxgs* had an effect on cell proliferation, the growth kinetics of *EhCoxgs* and control *Eh* were compared. We analyzed the growth of *Eh* over time and showed that during the first 24 h growth kinetics were similar, however at 48 and 72 h *EhCoxgs* proliferation was significantly slower than control *Eh* (Figure 1D). These results suggest that *Eh*Cox might be essential for cell growth and proliferation.

### *Eh*Cox Gene Silencing Caused Limited Proteome Change

To determine if *EhCoxgs* also affected the expression of other proteins, we took a proteomic approach and analyzed the proteome from *EhCoxgs* and control *Eh*. Only a limited number of proteins showed three-fold or higher expression (Supplementary Table 1). In *EhCoxgs*, 23 proteins were up regulated and 19 proteins were down regulated compared to control *Eh* (Table 2). *Eh*Cox protein (Q9U3Z8_ENTHI) was not detected in *EhCoxgs* proteome that confirms complete absence of the *Eh*Cox protein and gene specific silencing. Among the proteins that were up regulated included those encoding for several uncharacterized proteins (M7X297_ENTHI,A0A175JHX8_ENTHI,A0A175JJQ8_ENTHI,A0a175JU71_ENTHI,A0A175JQL9_ENTHI,A0A175LQ0_ENTHI,A0A175JJP8_ENTHI)and Rab family GTPase proteins (A0A175JL18_ENTHI,A0A175JT01_ENTHI,A0A175JSR0_ENTHI),UDP-glucose:glycoproteine glucosyltransferase (C4M0W6_ENTHI), WD domain containing protein (A0A175JMP8_ENTHI), Galactose specific adhesin light subunit (A0A175JFH2_ENTHI) and cysteine protease (A0A175JGF5_ENTHI) suggesting that increased expression of these genes particularly Rab family GTAPase and WD domain containing proteins that regulate vesicular trafficking of cysteine protease, may be involved in EhCox-mediated cysteine protease upregulation. The proteomics data showed a slightly increase in other cysteine proteases including CP1 and CP2 (Table 3). However, we did not detect *Eh*CP-A4 or *Eh*CP-A5 specifically in the dataset (Table 2, Supplementary Table 1). These observations led us to hypothesize that the increased expression and activity of CP5 and CP4 was not limited to a specific protease but rather, all the major CPs were regulated by *Eh*Cox. Similar to up regulated proteins we found several down regulated uncharacterized proteins (A0A175JKF9_ENTHI, C4LZW1_ENTHI, A0A175JJ73_ENTHI, A0A175JJ95_ENTHI). Other down regulated proteins were DNAj family protein (A0A175JFG6_ENTHI), Ras guanine nucleotide exchange factor (A0A175JYJ4_ENTHI), Ph domain containing protein (A0A175JQH4_ENTHI), and V-type proton ATPase subunit (A0A175JK72_ENTHI).

**Table 2:** List of genes that are up or down regulated ≥3-fold upon *Eh*Cox gene silencing.

**Protein name**	**Accession no**.	**Regulation**
Uncharacterized protein	M7X297_ENTHI	Up
Uncharacterized protein	A0A175JHX8_ENTHI	Up
UDP-glucose:glycoprotein glucosyltransferase	C4M0W6_ENTHI	Up
Uncharacterized protein	A0A175JJQ8_ENTHI	Up
tRNA cytosine 5 methyltransferase putative	A0A175JGM4_ENTHI	Up
Leucine-rich repeat containing protein	A0A175JM48_ENTHI	Up
Uncharacterized protein	A0A175JU71_ENTHI	Up
Uncharacterized protein	A0A175JQL9_ENTHI	Up
Uncharacterized protein	A0A175JLQ0_ENTHI	Up
Alpha-soluble nsf attachment protein putative	A0A175JLV9_ENTHI	Up
Serine-rich protein	A0A060N047_ENTHI	Up
Uncharacterized protein	A0A175JJP8_ENTHI	Up
Uncharacterized protein	A0A175JFS2_ENTHI	Up
Rab family GTPase	A0A175JLI8_ENTHI	Up
Uncharacterized protein	A0A175JRL0_ENTHI	Up
Uncharacterized protein	A0A175JV24_ENTHI	Up
LIM zinc finger domain containing protein	A0A060N6K9_ENTHI	Up
WD domain containing protein	A0A175JMP8_ENTHI	Up
Galactose-specific adhesin light subunit	A0A175JFH2_ENTHI	Up
Rab family GTPase	A0A175JT01_ENTHI	Up
Uncharacterized protein	A0A175JYV1_ENTHI	Up
tRNA (guanine-N(7)-)-methyltransferase non-catalytic subunit	A0A175K0F2_ENTHI	Up
Rab family GTPase	A0A175JSR0_ENTHI	Up
Cysteine protease	A0A175JGF5_ENTHI	Up
Uncharacterized protein	A0A175JKF9_ENTHI	Down
Uncharacterized protein	C4LZW1_ENTHI	Down
Uncharacterized protein	A0A175JJ73_ENTHI	Down
Uncharacterized protein	A0A175JJ95_ENTHI	Down
DNAj family protein	A0A175JFG6_ENTHI	Down
Ras guanine nucleotide exchange factor putative	A0A175JYJ4_ENTHI	Down
Uncharacterized protein	A0A175JL03_ENTHI	Down
Uncharacterized protein	A0A175K128_ENTHI	Down
Ph domain containing protein	A0A175JQH4_ENTHI	Down
V-type proton ATPase subunit	A0A175JK72_ENTHI	Down
70kDa heat shock protein	M2RGX4_ENTHI	Down
Spry domain protein	A0A175JUJ4_ENTHI	Down
Mov34 mpn pad 1 family protein	A0A175JY43_ENTHI	Down
Uncharacterized protein	A0A060N091_ENTHI	Down
Uncharacterized protein	A0A175JLR6_ENTHI	Down
Rho guanine nucleotide exchange factor putative	A0A175JXR9_ENTHI	Down
Casein kinase putative	A0A175JLP4_ENTHI	Down
Uncharacterized protein	A0A175JN68_ENTHI	Down
Type a flavoprotein putative	A0A175JLL6_ENTHI	Down

**Table 3:** Analyzed cysteine protease expression in *EhCoxgs* compared to control *Eh* by using total spectral counts.

**Identified proteins**	**Accession no**.	**Fold change**
Cysteine protease putative	A0A175JGF5_ENTHI	3.0
Cysteine proteinase 1	A0A060N046_ENTHI	1.3
Cysteine proteinase 2	A0A060N5V8_ENTHI	1.2
Cysteine proteinase 1	S0AV91_ENTHI	1.3

### Gene Silencing of *Eh*Cox Increases Cysteine Protease Expression and Activity

**Figure 2:**
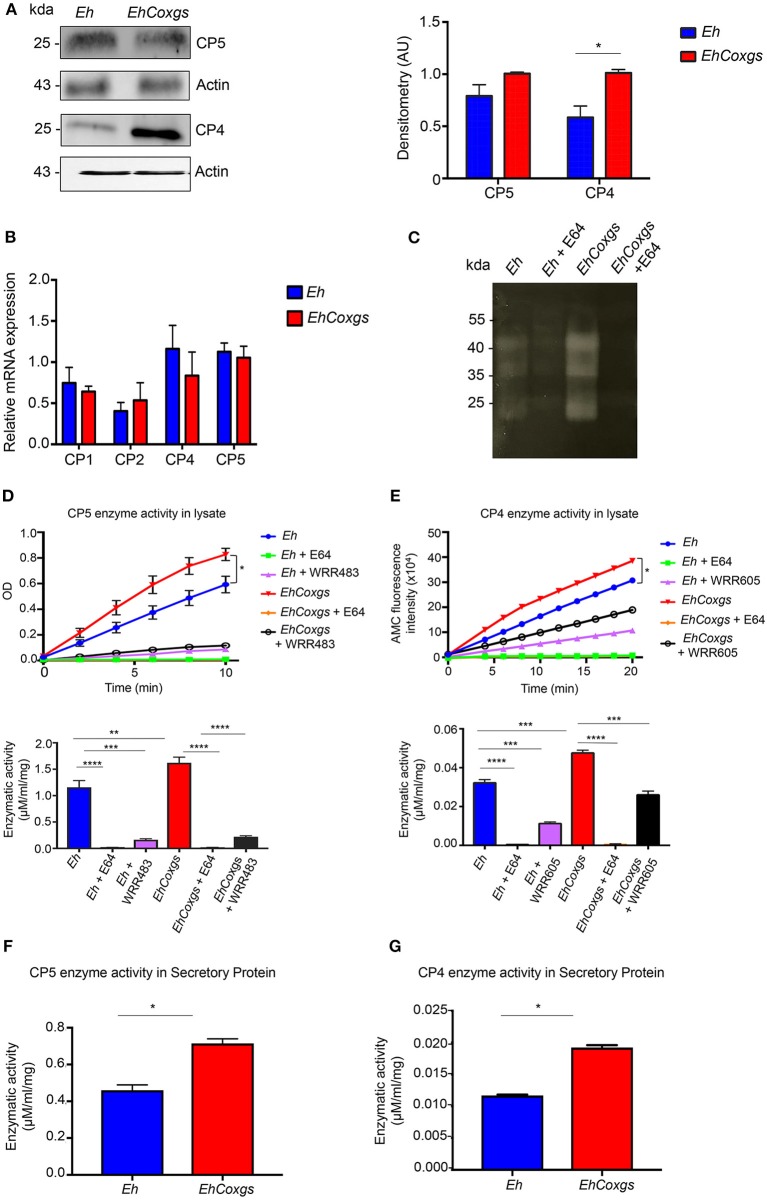
Gene silencing of *Eh*Cox increases cysteine protease expression and activity. **(A)** Immunoblot blot analysis was performed on lysate prepared from control *Eh* and *EhCoxgs*. The proteins were separated on 12% SDS PAGE gels and analyzed with CP5, CP4 or actin antibody. Quantifications of CP5/4 were performed by densitometric analysis from three independent experiments shown in right panel. **(B)** qPCR was used to monitor CPs expression using cDNA from *Eh* and *EhCoxgs*. Data indicate changes in mRNA expression compared with controls. **(C)** Gelatin substrate gel electrophoresis of lysate from control *Eh* and *EhCoxgs* treated/untreated with E64. **(D,E)** CPs enzymatic activity was evaluated by incubating inhibitor-treated/non-treated *Eh* with *Eh*CP-A5 or *Eh*CP-A4 substrates for 10 and 20 min, respectively and calculated in μM/min/mg, shown in the bottom panel. **(D)** *Eh*CP-A5 enzymatic activity in lysate with known substrates (Z-RR) and 20 μM of inhibitor WRR483. **(E)** *Eh*CP-A4 enzymatic activity in lysate with known substrates (Z-VVR) and 20 μM of inhibitor WRR605 and 100 μM E64. **(F)** *Eh*CP-A5 and **(G)** *Eh*CP-A4 enzymatic activity in secreted protein. The bars indicate the means and the error bars indicate the standard errors of the means for three different experiments. The asterisks indicate the results of comparisons with the controls. ^*^P < 0.05, ^**^P < 0.01, ^***^P < 0.002, ^****^P < 0.001.

Cysteine proteases play major roles in the pathogenesis of *Eh*. Specifically, *Eh*CP-A5 has been shown to be involved in tissue invasion by disrupting the protective mucus layer and stimulating pro-inflammatory response in colonic cells (Moncada et al., 2003; Hou et al., 2010). To determine the functional significance of *EhCox* derived PGE_2_ in *Eh* virulence, we analyzed the expression of *Eh*CPs protein and mRNA in *EhCoxgs*. Cell lysates from *EhCoxgs* and control *Eh* were assayed for CP expression by western blotting using *Eh*CP-A5 and *Eh*CP-A4 specific antibodies. These proteases are highly expressed and released extracellular basally and during infection (He et al., 2010; Kissoon-Singh et al., 2011). An unexpected finding was that *EhCoxgs* showed a slight increased in *Eh*CP-A5 and significantly up regulated *Eh*CP-A4 protein expression compared to control *Eh* (Figure 2A, Supplementary Figure 2). Surprisingly, we did not detect a corresponding increase in CPs transcripts by qPCR (Figure 2B) using primer specific for *CP1, CP2, CP4*, and *CP5* (Table 1). These results suggest that *Eh*Cox was regulating CPs at the translational level independent of transcription. To assess whether the increase in *Eh*CPs protein expression resulted in a corresponding increase in *Eh*CPs enzymatic activity, gelatin substrate gel electrophoresis was performed with *Eh* lysates that showed prominent bands of CPs activity in *EhCoxgs* as compared to control *Eh* lysate which was not present in E64 (CP inhibitor) treated *Eh* lysate (Figure 2C). To verify increased enzymatic activity, *EhCoxgs* and control *Eh* lysates were incubated with known substrates and proteinase activity was quantified by the liberation of chromogenic leaving group p-nitroanilide and the fluorescent leaving group 7-amino-4-methylcoumarin (AMC) from *Eh*CP5 and *Eh*CP4 peptide substrates, Z-Arg-Arg-pNA and Z-VVR-AMC, respectively. Degradation of *Eh*CPs substrate occurred in linear mode over time (Figures 2D,E) with significantly higher enzymatic activity in *EhCoxgs* as compared to control *Eh* lysates. Specificity for CPs enzymatic activity was confirmed using specific *Eh*CP-A5 and *Eh*CP-A4 inhibitors, WRR483 and WRR605, respectively (Figures 2D,E). *Eh*CP-A5 and *Eh*CP-A1 are unique as they both cleave the common substrate Z-Arg-Arg-pNA and here we used the *Eh*CP-A1 specific inhibitor WRR483 to show that *Eh*CP-A5 enzyme activity was specifically increased (St-Pierre et al., 2017). E64 inhibits all CPs in *Eh*. As *Eh*CPs are secreted extracellular we also found significantly increased *Eh*CP5/4 enzyme activity in *EhCoxgs* compared to control *Eh* (Figures 2F,G). These results clearly show that *EhCoxgs* expressed higher levels and activity of CPs, which was regulated by *Eh*Cox protein.

### Effect of Arachidonic Acid (AA), Aspirin and Prostaglandin E_2_ on Cysteine Protease Activity

**Figure 3:**
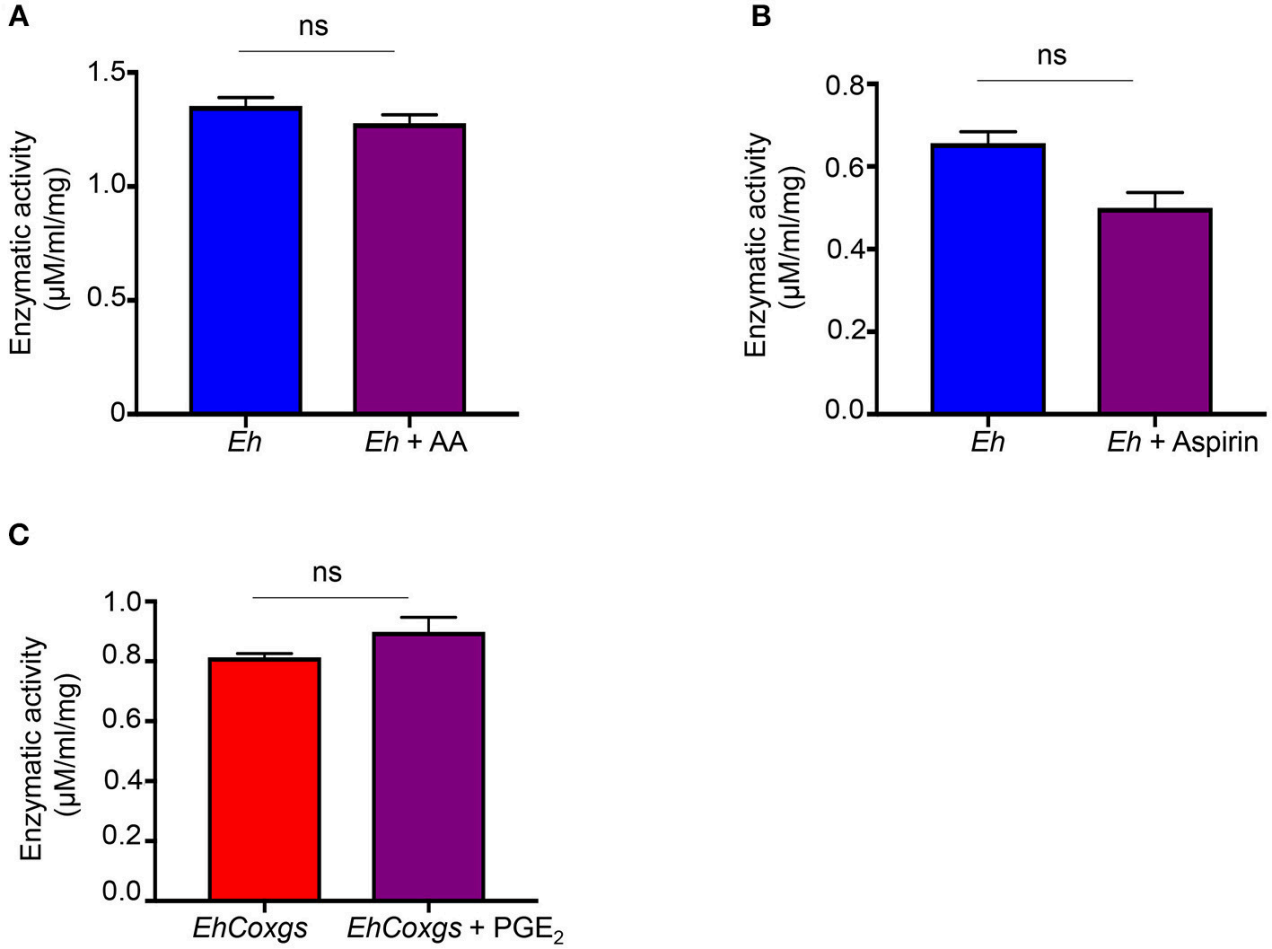
Effect of arachidonic acid, aspirin and prostaglandin on *Eh*CP-A5 cysteine protease activity. Live control *Eh* were pretreated with drugs for 16 h at 37°C. **(A)** Arachidonic acid (100 μM), **(B)** aspirin (1 mM) and **(C)** PGE_2_ (1 μM) for 16 h at 37°C. Lysates were prepared from drug treated/non-treated *Eh* using three cycles of freeze-thaw lysis. The bars indicate the means and the error bars indicate the standard errors of the means for three different experiments.

From the studies above it was unclear if inhibition of *Eh* PGE_2_ biosynthesis and/or Cox enzyme activity was regulating CP expression and enzyme activity in *EhCoxgs*. In live *Eh*, PGE_2_ is produced in a time-dependent manner in the presence of AA substrate (Dey et al., 2003). AA is the most important rate-limiting step in *Eh*Cox driven biosynthesis of PGE_2_ and aspirin is the only inhibitor known to inhibit *Eh*Cox enzymatic activity (Dey et al., 2003). To evaluate if PGE_2_ played a role in regulating *Eh*CP5 activity, control *Eh* was incubated with exogenous AA or aspirin and they had no effect on enzyme activity (Figures 3A,B). Similarly, the addition of exogenously prostaglandin (PGE_2_) to *EhCoxgs* showed no difference in *Eh*CP5 activity (Figure 3C). These results strongly suggest that the increase in *Eh*CP5/4 activity was not dependent on Cox enzymatic activity but rather appear to be a direct effect of the Cox protein on *Eh*CP expression and activity.

### *EhCoxgs* Stabilizes *Eh*CP-A5 Protein Degradation

**Figure 4:**
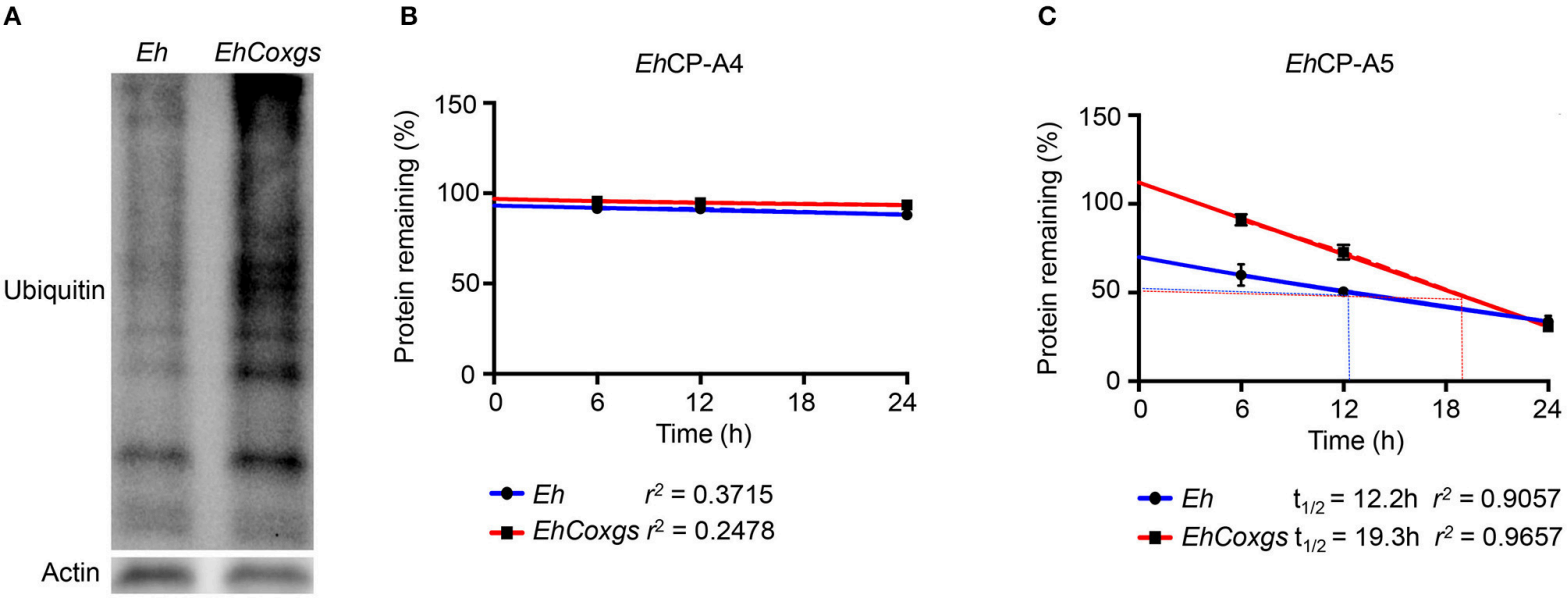
*EhCoxgs* stabilizes *Eh*CP-A5 protein degradation. **(A)** Log phase *Eh* and *EhCoxgs* proteins (30 μg) were loaded on a 12% SDS-PAGE gel and immunoblotted with an ubiquitin antibody (P4D1). Actin was used as a loading control. **(B,C)** The half-life of *Eh*CP-A4 and *Eh*CP-A5 protein was determined by treating *Eh* with cyclohexamide (see details in Materials and Methods) and the remaining CPs quantified by Western blot over 24 h. Protein half-life (${\text{t}}_{\frac{1}{2}}$) was calculated using regression analysis.

To address the mechanism whereby *EhCoxgs* increased protein expression and enzymatic activity, we hypothesize that the CPs in *EhCoxgs* were not degraded based on increased protein expression (Figure 2A). As several proteins were up/down regulated in *EhCoxgs* (Table 2), we were surprised that more proteins were ubiquitinated and destine for 26S proteasome degradation in *EhCoxgs* as compared to *Eh* (Figure 4A). Based on these findings we theorize that proteins critical in regulating the stability of *Eh*CPs maybe degraded and quantified the half-lives of both *Eh*CP-A4/5. To do this, *Eh* and *EhCoxgs* were treated with cycloheximide and the percentage protein remaining over 24 h determined. Surprisingly, *Eh*CP-A4 protein was not degraded whereas the half-life for *Eh*CP-A5 was 19.3 h in *EhCoxgs* as compared to 12.2 h for control *Eh* (Figures 4B,C). The increase in *Eh*CP-A5 protein stability in *EhCoxgs* may account for increase protein accumulation and increase enzyme activity. In contrast, *Eh*CP-A4 protein was very stable and turnover rate low. We did not treat *Eh* longer than 24 h with cyclohexamide (95% viable by trypan blue exclusion) as cells became rounded and detached from the glass tubes and we were concern about cell death.

### Increased Cysteine Protease Expression Results in Increased Erythrophagocytosis and Cytopathic Activity

**Figure 5:**
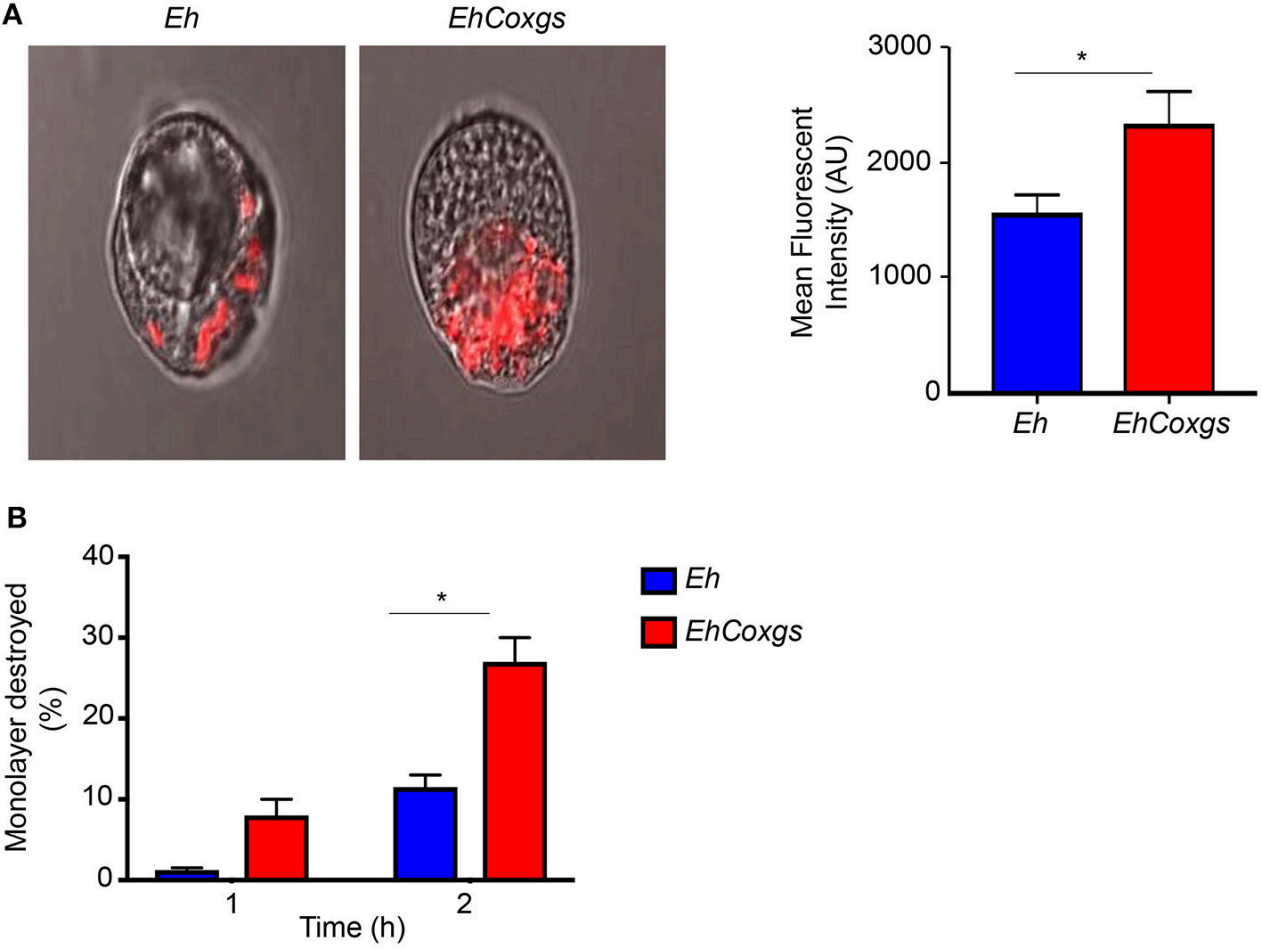
Effect of *EhCox* gene silencing on erythrophagocytosis and cytopathic activity. **(A)** Confocal microscopy of Eh after 20 min incubation with Phicoerythrin labeled RBC at 37°C. Quantitation of erythrophagocytosis was performed with mean florescent intensity (MFI) analysis from three independent experiments, AU, arbitrary units. **(B)** *EhCoxgs* showed increase Caco-2 cells monolayer destruction in comparison to control *Eh* in a time-dependent manner. The bars indicate the means and the error bars indicate the standard errors of the means for different experiments. The asterisks indicate the results of comparisons with the controls. ^*^P < 0.05.

Experimental evidence suggests a direct correlation between *Eh* virulence and the rate of phagocytosis and protease activity (Ankri et al., 1998, 1999; Okada et al., 2005; Hirata et al., 2007). Erythrophagocytosis is considered as one of the prominent marker of *Eh* virulence (Trissl et al., 1978; Orozco et al., 1983; Bhattacharya et al., 2002). Accordingly, we assayed the ability of *EhCoxgs* to phagocytose fluorescently labeled RBCs and measured the fluorescence intensity by confocal microscopy. *EhCoxgs* showed significantly higher RBCs uptake in comparison to control *Eh* (Figure 5A). *EhCoxgs* also significantly destroyed 27% of a Caco-2 monolayer after 2 h incubation compared to control *Eh* that destroyed 11% (Figure 5B). These results show that *EhCoxgs* is highly phagocytic with increased cytopathic activity.

### Differential Math1 Transcriptional Activity in Math1^GFP^ Mice Exposed to *EhCoxgs*

**Figure 6:**
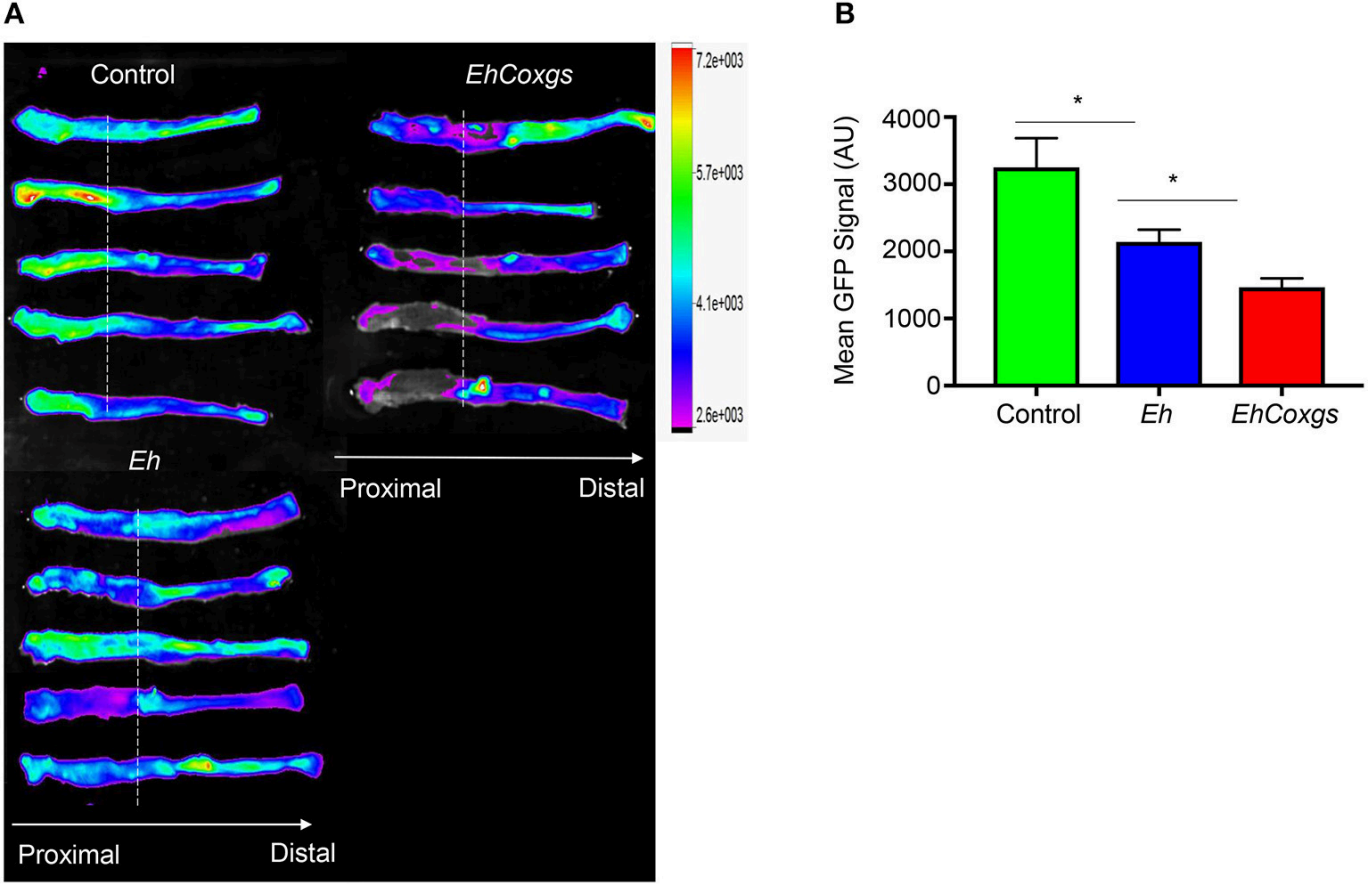
Differential Math1 transcriptional activity in Math1 GFP mice exposed to *EhCoxgs* due to increased cysteine proteases activity. **(A)** Math1 expression heatmap in Math1^GFP^ mice colons, dotted line indicates the colonic loop ligation. **(B)** GFP signal quantification in the proximal colon. GFP, green fluorescent protein; AU, arbitrary units. ^*^P < 0.05.

Based on the results above, we then determined if there was a similar increase in *EhCoxgs* virulence using closed colonic loops in mice (Kissoon-Singh et al., 2013). We have previously shown that *Eh* induces hyper secretion of mucus by goblet cells and elicits an acute pro-inflammatory response in colonic loops (Dharmani et al., 2009). In particular, *Eh*CP5 RGD motif has been shown to bind αvβ3 integrin on goblet cells to elicit mucin hyper secretion (Cornick et al., 2016). We hypothesized that increase CPs activity would result in a differential response toward mucin biosynthesis and secretion to *EhCoxgs* as compared to control *Eh*. To interrogate this, we used Math1^GFP^ mice containing the green fluorescent protein (GFP) reporter for Math1-expressing goblet cells. In the colon, Math1 is expressed in epithelial cells to differentiate into Muc2-producing goblet cells lineage. Basally, Math1^GFP^ activity was higher in the proximal colon in control mice. However, following *Eh* infection, Math1 activity was significantly decreased in the proximal colon, which was decreased further when mice were infected with *EhCoxgs* (Figure 6). We have previously shown that decrease in Math1^GFP^ activity correlates with increase pro-inflammatory activity in response to DSS-induced colitis (Tawiah et al., 2018).

### Pro-inflammatory Responses Are Exacerbated in Math1 Mice Exposed to *EhCoxgs* Compared Control *Eh*

**Figure 7:**
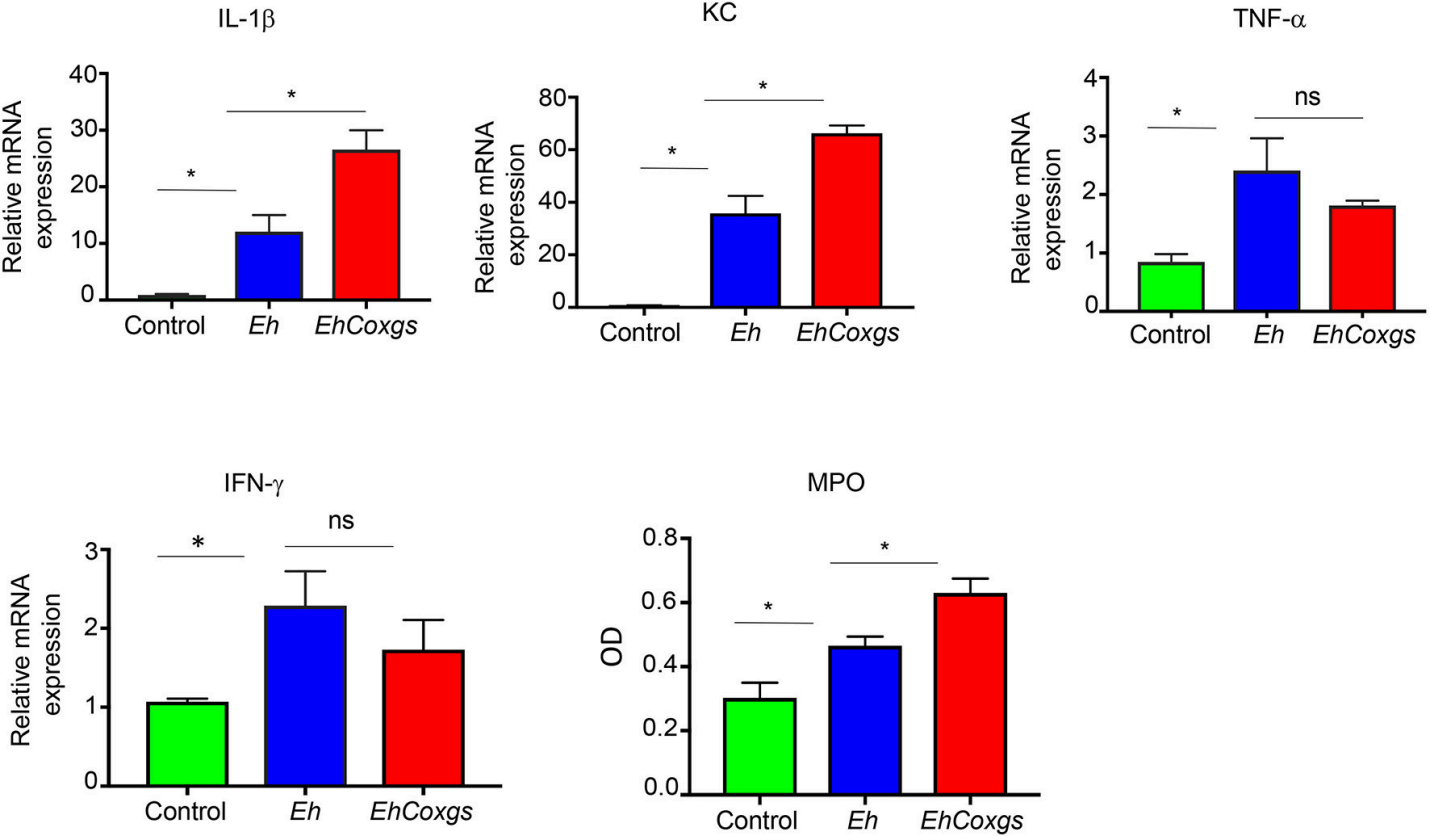
Pro-inflammatory responses are exacerbated in Math1 mice exposed to *EhCoxgs* compared control *Eh*. Math1^GFP^ mice were infected with control *Eh* or *EhCoxgs* for 3 h. Gene expression of IL-1β, KC, TNF-α, and IFN-γ was examined from excised tissues from the proximal colon. Myeloperoxidase activity in the proximal colons of mice inoculated with control *Eh* or *EhCoxgs*. The bars indicate the means and the error bars indicate the standard errors of the means for different experiments. ^*^P < 0.05.

It is well-known that *Eh* infection in the gut elicits an acute pro-inflammatory response with the production of pro-inflammatory cytokines IL-1β, IL-8, IFN-γ, and TNF-α (Bansal et al., 2009; Galván-Moroyoqui et al., 2011). Based on the results above showing a marked decrease in Math1^GFP^ activity in the proximal colon, we hypothesize that enhanced pro-inflammatory cytokines were suppressing Math1 activity. Indeed, pro-inflammatory cytokines expression in colonic tissues after 3 h exposure with *EhCoxgs* showed increased expression of IL-1β and KC (human IL-8 homolog) but not TNF-α and IFN-γ as compared to *Eh*-inoculated loops (Figure 7). Both IL-1β and KC are released by epithelial and immune cells recruited to the site of infection that can exacerbate tissue injury (Mortimer and Chadee, 2010). Specifically, KC is a potent neutrophil chemo attractant that recruits neutrophils to the site of infection but is ineffective in clearing the parasite. Pro-inflammatory cytokine expression correlated with increased myeloperoxidase (MPO) activity in response to *EhCoxgs* as compared to control *Eh* (Figure 7). These results reveal that *EhCoxgs* with increased CP enzyme expression and activity enhanced virulence in the mouse colon to elicit increased pro-inflammatory responses.

## Discussion

In this study, we describe how *Eh* cysteine protease expression and activity is regulated by another enzyme, cyclooxygenase (Cox). Cox is the critical rate limiting step in the biosynthesis of PGE_2_ (Smith and Marnett, 1991; Vane et al., 1998). Cox was identified in *Eh* that showed little homology to known Cox1/2 enzyme across different species with absence of conserved arachidonic acid binding domain and catalytic site that are present in other species. However, endogenous and recombinant *Eh*Cox showed Cox like enzymatic activity by using arachidonic acid as substrate that was inhibited only by aspirin but not by another Cox-1/2 inhibitors (Dey et al., 2003). In a later study, Cox like protein (Acc No. AF208390) was characterized as an actinin like protein in *Eh* as it has actin- and calcium-binding domains. The actin binding domain of *Eh* actinin like protein share only 30% identity with other actin binding proteins however, the protein showed 28% sequence identity to *D. discoideum* actinin protein. Based on some of the unusual domain architecture feature of *Eh* actinin like proteins it was proposed that this unusual protein might differ in function from known actinin proteins (Heike et al., 2005). Beside actin bundling, multiple cellular functions of the actinin protein have been proposed with putative interacting partners (Otey and Carpen, 2004; Cabello et al., 2007). For example, mammalian actinin regulates several receptor activity by linking the cytoskeleton to a variety of trans membrane proteins (Cabello et al., 2007). From these studies and our findings, we proposed that besides Cox like activity and actin polymerization function, this protein might have multifunctional roles in the regulation of several other proteins in *Eh*. Based on its molecular structure and proposed multiple functions we developed *EhCoxgs* to determine its biological function in the pathogenesis of amebiasis.

Cysteine proteases (CPs) are ubiquitous and are differentially regulated in virulent and non-virulent strains of *Eh*. Previous studies have showed that CPs are key virulent factors in the pathogenesis of *Eh* that are released during tissue invasion and one of the most important protein involved in phagocytosis (Okada et al., 2005; Hirata et al., 2007). In the present study, we have shown that silencing the Cox gene increased cysteine protease expression and activity endogenously and extracellular without affecting CPs transcript. Increased cysteine protease activity led us to hypothesize that Cox derived PGE_2_ was negatively regulating cysteine protease activity. However, the addition of exogenous arachidonic acid to live *Eh* to increase PGE_2_ production or aspirin to inhibit PGE_2_ or exogenous PGE_2_ did not affect CP activity. These observations suggest that increase in CPs expression and activity was not the effect of Cox enzymatic activity/end product of biosynthesis but rather was regulated by the Cox protein itself. The *Eh*Cox protein acted as a negative regulator of CPs. Since expression of CPs was not regulated at the transcriptional level, there is the possibility of post-translational modification of CPs up regulating its expression and enzymatic activity. In support of this we have shown increased *Eh*CP-A5 protein stability in *EhCoxgs* as compared to control *Eh*. While the mechanism of this post-translational regulation is not known, our results suggest that proteins critically involved in regulating *Eh*CP-A5 turnover were degraded in *EhCoxgs* by the appearance of more ubiquitinated proteins. In contrast, *Eh*CP-A4 protein was stable and turnover rate low over 24 h that suggest different regulatory mechanisms between the CPs. No doubt uncovering the post-translational regulation of CPs enzyme will provide the basis to understand the mechanism of Cox mediated regulation and promote the development of more efficient therapeutic strategies of indirectly targeting CPs enzyme.

Proteomic analysis of the *EhCoxgs* revealed high expression of Rab family GTAPase and WD domain containing proteins as compared to control *Eh*. These proteins are involved in various cellular process including membrane trafficking, cytoskeletal assembly and cell proliferation (Saito-Nakano et al., 2005; Nakada-Tsukui and Nozaki, 2015). Based on this observation, we proposed that these proteins might be involved in up regulating CPs when *Eh*Cox is silenced. However, it needs to be further determined what functional advantages or constraints drive CPs up regulation. We also found iron-sulfur falvoprotein (A0A175JR31, M7W6A3; Supplementary Table 1) among the down regulated proteins in *EhCoxgs* which supports reduced growth of *EhCoxgs* as these proteins have been shown to be essential for the growth and survival of *Eh* under different condition (Nozaki et al., 1998; Shahi et al., 2016). However, the question that remains to be answered is the mechanism of how *Eh*Cox regulates *Eh* growth.

Phagocytosis is an active process in *Eh* and prominent marker of parasite pathogenicity. Phagocytosis involves several steps and activation of signaling pathways (Orozco et al., 1983; Hirata et al., 2007). A proteomic study of phagosome showed the involvement of several proteins in this process including cysteine protease and vesicular transport proteins (Okada et al., 2005). Since more cysteine protease (activity assay and expression) and several vesicular trafficking proteins (proteomics analysis) were observed in *EhCoxgs*, we analyzed the phagocytosis capacity of *EhCoxgs* as compared to control *Eh*. Unexpectedly, we found increased erythrophagocytosis and cytopathic activity in *EhCoxgs* as compared to control *Eh*. While it is difficult to explain how increased in *Eh* CPs can enhance phagocytosis, these data are consistent with previous studies that showed CPs are directly involved in destruction of colonic epithelial cells, tissue invasion, phagocytosis and degrade host antibodies and complement in immune evasion and disease pathogenesis (Hirata et al., 2007; Mortimer and Chadee, 2010; Nakada-Tsukui and Nozaki, 2016).

Several *in vivo* and *ex vivo* assays reported the recruitment of inflammatory cell in *Eh*-induced infection/lesion as a consequence of localized expression of chemokines and cytokines at the site of infection, leading to an inflammatory response (Bansal et al., 2009; Kissoon-Singh et al., 2013; Nakada-Tsukui and Nozaki, 2016). To address whether *EhCoxgs* were more virulent, we used closed colonic loop as short infection model to quantify acute inflammatory responses. Our study revealed that the Cox protein played a major role in *Eh*- induced fluid secretions and pro-inflammatory cytokine responses by regulating CPs activity. In particular, *EhCoxgs* elicited high levels of IL-1β and KC expression demonstrating cysteine protease dependent induction of IL-1β and IL-8 expression as observed in *Eh* infected SCID mouse-human intestinal xenograft (Seydel et al., 1997). Previous studies have shown CP5 to be important in enhancing mucosal inflammation by cleaving the released pro-IL-1β into its active form and inducing IL-8 expression in mast cell (Zhang et al., 2000; Lee et al., 2014). Both TNF-α and IFN-γ were up regulated in response to *Eh* and was not enhanced further by *EhCoxgs*. In *EhCoxgs* inoculated colonic loops we also found elevated MPO activity, a marker for increased flux of neutrophils that are responsible for exacerbating tissue injury.

Increased CP expression and virulence in *EhCoxgs* is intriguing which led us to propose the following hypotheses. First, *Eh*Cox is as endogenous inhibitor of cysteine proteases. While examples of identified cysteine proteinase inhibitors produced by parasites are rare they can be targeted to treat disease related to increased protease activity. For example, papain inhibitors were detected in parasitic protozoa including *Leishmania, Trichomonas*, and *Trypanosoma*, suggesting existence of these inhibitors are widespread (Irvine et al., 1992). In *Schistosoma mansoni*, a gene was identified which encode papain inhibitors (Cao et al., 1993). In *Eh*, few CP inhibitors have been characterized that negatively regulates CP secretion and thus, virulence of the parasite (Sato et al., 2006). Second, CP regulation by Cox is a negative feedback mechanism to reduce host inflammation. This negative feedback regulation may counteract excessive cysteine protease function at site of inflammation and thus, decrease the likelihood of tissue damage that led to amebic lesion/colitis. This phenomenon may furthered explain why most *Eh* infections are asymptomatic. Consistent with this hypothesis, a study in mice showed that ribosomal protein S19 interact with macrophage migration inhibitory factor and function as an extracellular inhibitory factor by attenuating its pro-inflammatory function (Filip et al., 2009). Another study in *Arabidopsis thaliana* proposed the formation of a complex network of cysteine protease-Serpin1 interaction controlling innate immunity during plant development (Rustgi et al., 2017). Based on these findings it would be interesting to determine whether these proteins can interact together; it is likely these interactions could regulate CPs activity either as a result of conformational changes of the enzyme or by impacting subcellular localization of CPs and thus affecting its interactions with Cox, to further modulate its activity. Furthermore, the effect of this interaction on activity and expression of cysteine protease or vice versa can be analyzed. This information can be used to develop chemotherapeutic target against *Eh* infection. Clearly further studies are needed to understand the underlying molecular mechanisms by which *EhCoxgs* increases CPs expression and activity.

## Author Contributions

PS and KC conceived and design the experiments and wrote the manuscript. PS and FM performed the experiments. PS analyzed the data.

### Conflict of Interest Statement

The authors declare that the research was conducted in the absence of any commercial or financial relationships that could be construed as a potential conflict of interest.

## Supplementary Material

Supplementary Table 1 Entire proteomics profile of control *Eh* and *EhCoxgs* with mass spectrometry counts, accession number and protein ID.

Supplementary Figure 1 Full scan of blots for Cox protein shown in Figure 1B.

Supplementary Figure 2 Full scan blots of CP5/4/actin shown in Figure 2A.
