# Supplementary figures and images for: Entamoeba histolytica Cyclooxygenase-Like Protein Regulates Cysteine Protease Expression and Virulence
Source: Front Cell Infect Microbiol. 2019 Jan 9;8:447. doi: 10.3389/fcimb.2018.00447 (PMC6333869; doi:10.3389/fcimb.2018.00447)

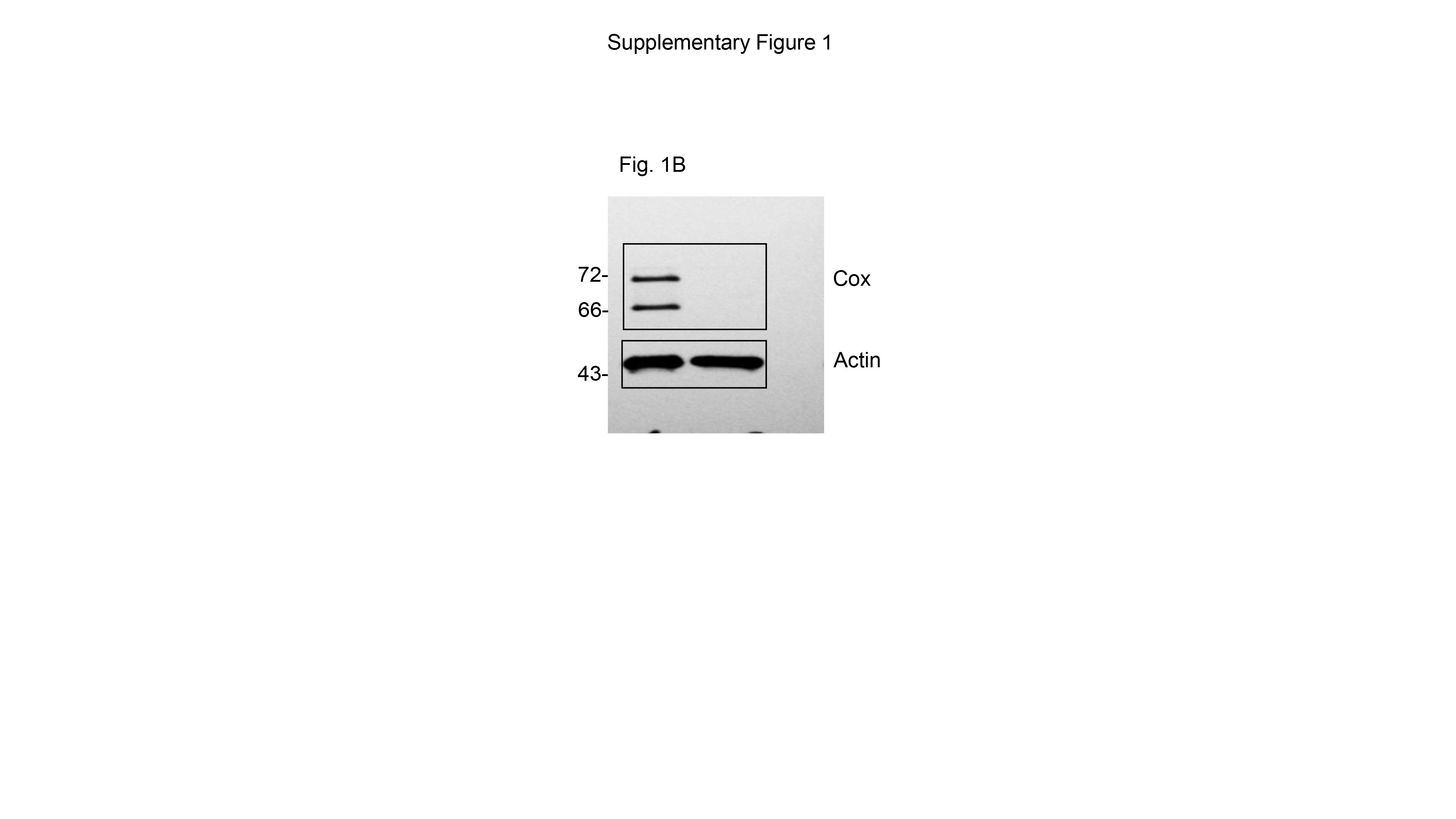

Supplement: Supplementary Figure 1 — Full scan of blots for Cox protein shown in Figure 1B. [file Image_1.JPEG]

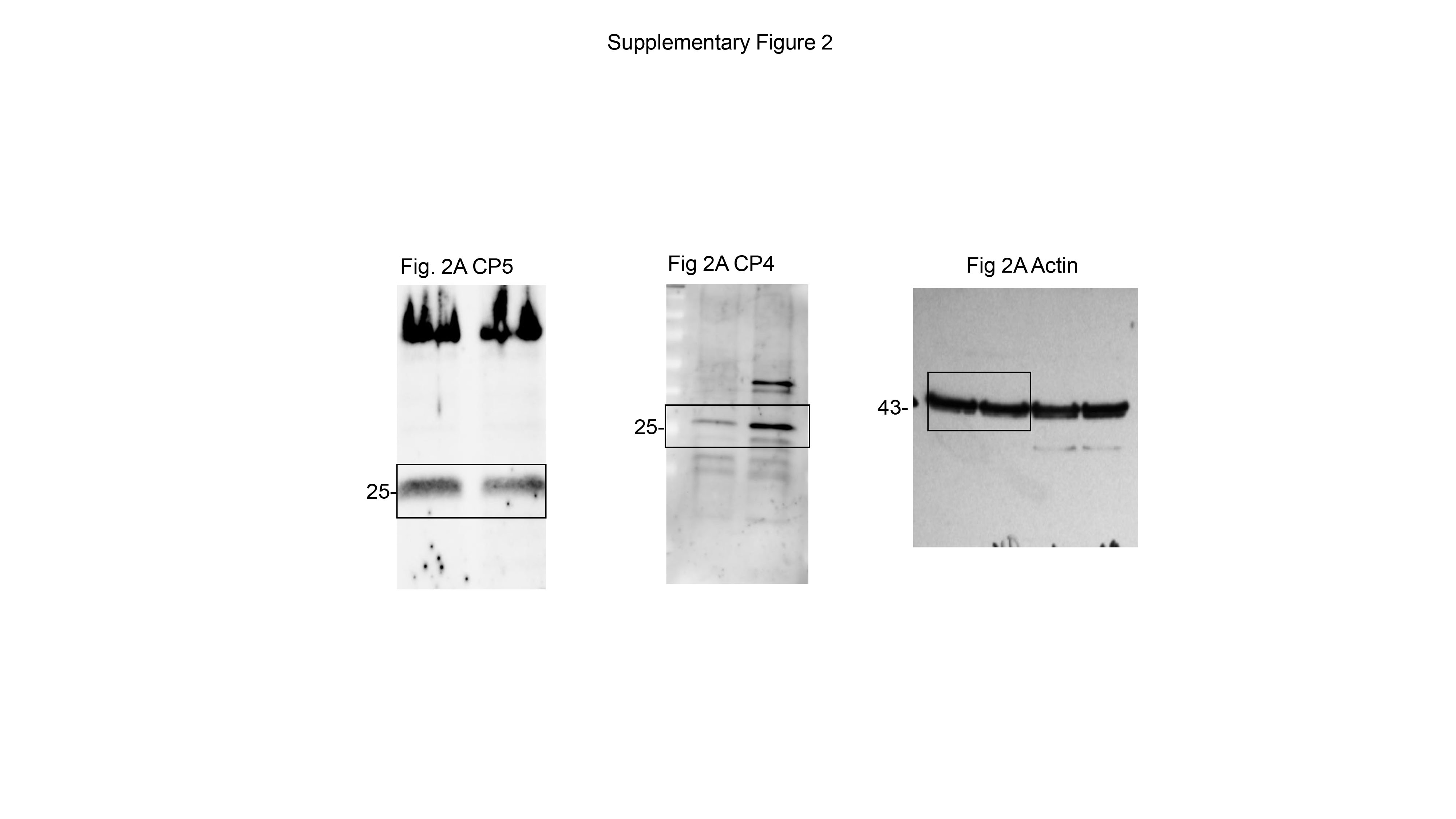

Supplement: Supplementary Figure 2 — Full scan blots of CP5/4/actin shown in Figure 2A. [file Image_2.JPEG]
